# Diclofenac and Meloxicam Exhibited Anti-Virulence Activities Targeting Staphyloxanthin Production in Methicillin-Resistant *Staphylococcus aureus*

**DOI:** 10.3390/antibiotics12020277

**Published:** 2023-01-31

**Authors:** Rana A. Elmesseri, Sarra E. Saleh, Sarah A. Ghobish, Taghreed A. Majrashi, Heba M. Elsherif, Khaled M. Aboshanab

**Affiliations:** 1 Department of Microbiology, Faculty of Pharmacy, Misr International University (MIU), Cairo 19648, Egypt; 2 Department of Microbiology & Immunology, Faculty of Pharmacy, Ain Shams University (ASU), Cairo 11566, Egypt; 3 Department of Chemistry, Faculty of Pharmacy, Misr International University (MIU), Cairo 19648, Egypt; 4 Department of Pharmacognosy, College of Pharmacy, King Khalid University, Abha 61421, Saudi Arabia

**Keywords:** saphyloxanthin, MRSA, diclofenac, meloxicam, anti-virulence

## Abstract

*Staphylococcus aureus* (*S. aureus*) is a worldwide leading versatile pathogen that causes a wide range of serious infections. The emergence of antimicrobial resistance against *S. aureus* resulted in an urgent need to develop new antimicrobials in the new era. The methicillin-resistant *S. aureus* (MRSA) prevalence in hospital and community settings necessitates the discovery of novel anti-pathogenic agents. Staphyloxanthin (STX) is a key virulence factor for the survival of MRSA against host innate immunity. The current work aimed to demonstrate the anti-virulence properties of meloxicam (MXM) as compared to diclofenac (DC), which was previously reported to mitigate the virulence of multidrug-resistant *Staphylococcus aureus* and test their activities in STX production. A total of 80 *S. aureus* clinical isolates were included, wherein a qualitative and quantitative assessment of STX inhibition by diclofenac and meloxicam was performed. The quantitative gene expression of STX biosynthetic genes (*crt*M, *crt*N and *sig*B) and *hla* (coded for α-hemolysin) as a virulence gene with and without DC and MXM was conducted, followed by molecular docking analysis for further confirmation. DC and MXM potently inhibited the synthesis of STX at 47 and 59 µg/mL to reach 79.3–98% and 80.6–96.7% inhibition, respectively. Treated cells also revealed a significant downregulation of virulence genes responsible for STX synthesis, such as *crt*M, *crt*N and global transcriptional regulator *sig*B along with the *hla* gene. Furthermore, computational studies unveiled strong interactions between the CrtM binding site and DC/MXM. In conclusion, this study highlights the potential role and repurposing of DC and MXM as adjuvants to conventional antimicrobials and as an anti-virulent to combat MRSA infections.

## 1. Introduction

*Staphylococcus aureus* (*S. aureus*) is one of the most predominant Gram-positive bacteria responsible for a wide range of bacterial infections, including bacteremia, pneumonia, osteomyelitis, skin infections, and heart valve infections [1]. Antibiotic abuse in healthcare settings triggered the emergence of methicillin-resistant *S. aureus* (MRSA) in 1961 [2]. According to the Centers for Disease Control and Prevention (CDC), treatment approaches have become progressively challenging since the dawn of MRSA [3]. In 2017, the World Health Organization (WHO) reported MRSA to be a high-priority multidrug-resistant (MDR) pathogen [4]. The abundance of virulence factors produced by MRSA supports its survival and persistence under unfavorable conditions in vivo. Anti-infective therapy that targets virulence factors has recently been considered a suitable approach for combating the pressing matter of antibiotic resistance as well as the scarcity of novel antibiotics [5]. 

Despite the vast production of virulence determinants by MRSA, Staphyloxanthin (STX) is the most renowned for being only produced by *S. aureus* strains and is accountable for the naming of *S. aureus* [6]. STX is crucial for MRSA survival against the host’s innate immune defenses. The carotenoid pigment conveys resistance to MRSA by acting as an antioxidant to fight reactive oxygen species (ROS) and host neutrophil-based killing in addition to its ability to scavenge free radicals, hydrogen peroxide, singlet oxygen, and hypochlorous acid [7,8]. Previous studies involving mouse models divulged the positive correlation between STX production and antioxidant activity, where the pigmented strains of *S. aureus* were more resistant to neutrophil-based killing than non-pigmented strains [9]. Consecutively, inhibiting STX production is recognized as an effective anti-infective approach for hindering MRSA pathogenicity [10]. STX pigment is synthesized by an array of enzymes encoded by the *crtOPQMN* operon. The biosynthetic pathway of STX starts with the catalysis of the condensation of two farnesyl diphosphates to produce presqualene diphosphate by dehydrosqualene synthase (CrtM). Intriguingly, the STX biosynthetic pathway in *S. aureus* shares the first step with the human cholesterol biosynthesis pathway due to a structural similarity between CrtM and human squalene synthase (SQS). This resemblance in structure between CrtM and SQS led to the repurposing of some cholesterol-lowering agents as an anti-virulence therapy against pathogenic *S. aureus* through their interaction with CrtM [11]. The subsequent step in the STX biosynthetic pathway is catalyzed by 4,4-diapophytoene desaturase (CrtN), which converts 4,4-diapophytoene to 4,4-diaponeurosporene and had been proven as an endorsed target for anti-virulence therapy [12]. Furthermore, 1,4-Benzodioxan-Derivatives, Naftifine, Terbinafine and compound NP16 have been effectively recognized as potential CrtN inhibitors and hence STX inhibitors [13,14]. 

In an attempt to uncover novel anti-virulence drugs, previous studies have tested non-steroidal anti-inflammatories (NSAIDs) as new molecules targeting virulence factor inhibition that mitigate the virulence of multidrug-resistant *Staphylococcus aureus.*

As previously reported by Abbas et al., diclofenac (DC) was able to mitigates the virulence of multidrug-resistant *Staphylococcus aureus* and has inhibitory activities against STX production [15]. Several studies suggest that diclofenac could hinder the proliferation of various micro-organisms such as *S. aureus*, *Escherichia coli, Candida albicans, Mycobacterium tuberculosis,* and *Listeria monocytogenes* [16,17,18]. Likewise, a previous study proposed that Meloxicam (MXM) could enhance the susceptibility of *Pseudomonas aeruginosa* biofilm to antimicrobials [19]. Supporting previous studies in the field of anti-virulence therapy, the current work aimed to investigate the potential role of MXM as an anti-virulence agent targeting STX inhibition in MRSA isolates and compare its activity to that of DC as a promising drug repurposing approach for combating antibiotic resistance. 

## 2. Results

### 2.1. Identification of Clinical Isolates 

All Staphylococcal isolates revealed a characteristic grape-like cluster appearance by Gram stain, confirming the morphology of *S. aureus*. The clinical isolates produced yellow colonies on the MSA. Out of the 80 isolates, 57 isolates (71%) showed beta-hemolysis on blood agar, and 23 isolates (29%) showed no hemolysis on blood agar. Further biochemical identification of the species level revealed that all isolates were catalase- and coagulase-positive as well as DNase producers. The highest percentage of isolates were recovered from blood specimens (40%), followed by nasal swabs, wound swabs, pus, and groin swabs, with percentages 22.5%, 17.5%,12.5% and 7.5%, respectively.

### 2.2. Antibiotic Resistance Pattern 

As shown in Table 1, 100% of the isolates were resistant to cefoxitin. For clindamycin and ciprofloxacin, MRSA isolates showed a resistance pattern of 36.25% and 41.25%, respectively. However, 100% of the isolates were sensitive to vancomycin as well as linezolid. Multidrug resistance phenotype was evident in 30% of the isolates, as these isolates showed resistance to three antimicrobial agents of different classes (cefoxitin, clindamycin and ciprofloxacin).

### 2.3. Staphyloxanthin Production Assay 

**Figure 1:**
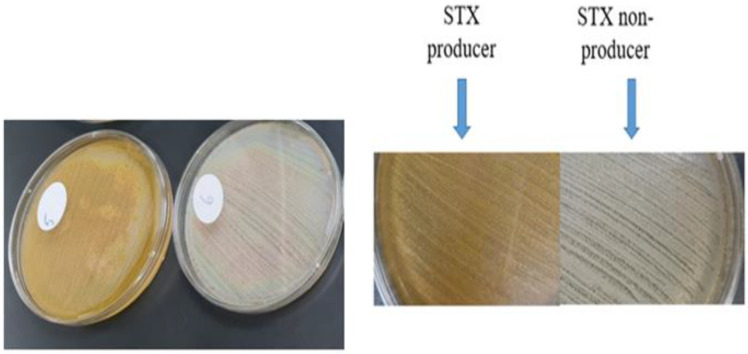
STX production in pigmented isolates versus non-pigmented isolates.

The pigmented MRSA isolates revealed a golden-yellow pigment after 48 h of incubation, indicating a high potential for producing STX. On the other hand, non-pigmented isolates failed to produce the golden pigment indicating their incapability of producing STX (Figure 1).

### 2.4. Quantitative Estimation of Staphyloxanthin Production

**Figure 2:**
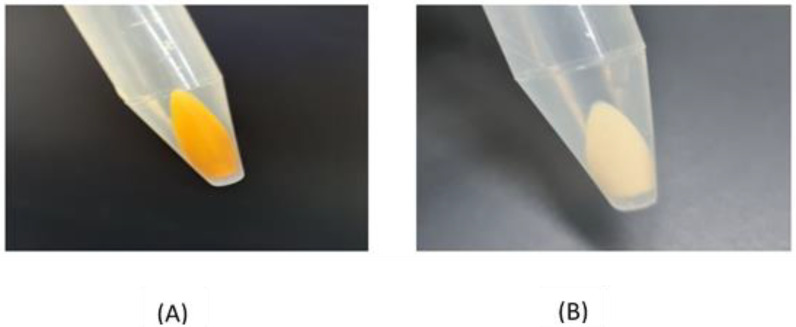
(**A**) Photographed pelleted cells of a pigmented isolate; (**B**) photographed pelleted cells of a non-pigmented isolate.

The STX production was quantified by measuring the absorbance of the methanolic extracts of the tested isolates spectrophotometrically at 450 nm (Figure 2). The isolates were categorized based on their optical densities (OD) into strongly pigmented (OD_450_ > 0.2), weakly-pigmented (OD_450_ = 0.1–0.2) and non-pigmented isolates (OD_450_ < 0.1). Out of the recovered isolates, 31 (38.75%) were found to be strongly pigmented and 19 (23.75%) were weakly pigmented, while 30 (37.5%) were non pigmented isolates. The standard strain MRSA ATCC 43300 and the Wild type (W.T) *S. aureus* proved to show the highest potential for pigmentation, as they showed the highest absorbance—0.779 and 0.771, respectively.

### 2.5. Determination of MICs of Tested Drugs 

The MICs of DC and MXM were determined for pigmented isolates (N = 31), standard strain MRSA and Wild type (W.T) MRSA. The results revealed that 375 µg/mL was the lowest concentration of DC, showing no visible growth in 61.9% of the isolates, whereas 973.5 µg/mL was the MIC for MXM in 80.6% of the isolates. Trials using the sub-MICs of the tested drugs (1/4, 1/8 and 1/16) were performed on selected strongly pigmented isolates. The results indicate that the sub-MICs (47 µg/mL) of DC and (59 µg/mL) of MXM did not interfere with the growth of the MRSA isolates, as no significant difference was observed between treated and untreated isolates, indicating that the sub-MICs had no bactericidal activity.

### 2.6. The Effect of the Tested Drugs on Staphyloxanthin Synthesis 

**Figure 3:**
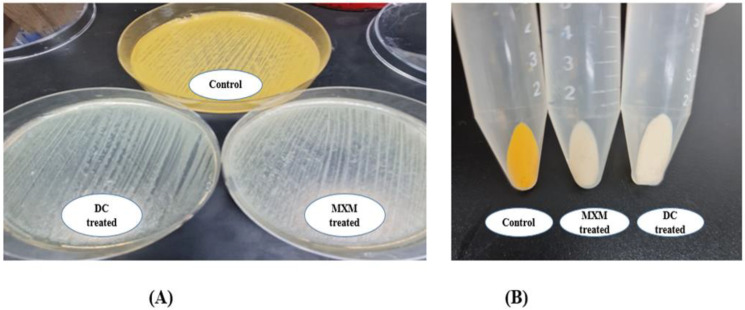
(**A**) Qualitative assessment of DC/MXM treatment on STX production; (**B**) quantitative assessment of DC/MXM treatment on STX production.

The influence of DC and MXM on STX production was examined in strongly pigmented and weakly pigmented MRSA isolates. The OD_450_ of the methanolic extracts of treated MRSA isolates was notably decreased after DC/MXM treatment when compared to their controls (Figure 3).

**Figure 4:**
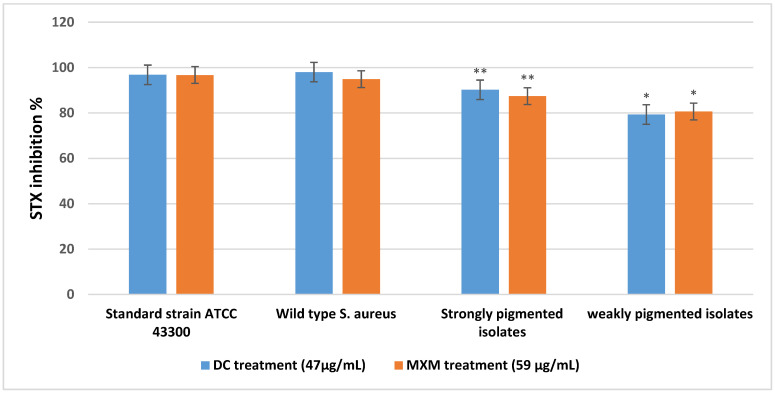
Mean and standard deviation values for STX inhibition percentage of strongly and weakly pigmented isolates in comparison with the standard strain and wild type *S. aureus.* ** represents higher statistical significance difference among strongly pigmented isolates than weakly pigmented isolates (*) with both drugs at *p* ≤ 0.05.

As shown in Figure 4, STX production was significantly inhibited by DC (at a concentration of 47 µg/mL, reaching 98%, 96.8%) and by MXM (at a concentration of 59 µg/mL, reaching 94.9%, 96.7%) in the W.T and standard strain, respectively. The strongly pigmented isolates category revealed comparable results of 90.2% STX inhibition when treated with DC and 87.4% when treated with MXM. The weakly pigmented isolates were less affected by the treatment; STX inhibition was recorded to be 79.3% and 80.6% with DC and MXM, respectively. With both drugs, there was a statistically significant difference between the percentage of STX inhibition among strongly pigmented and weakly pigmented isolates, with *p*-values < 0.001 and 0.007 for DC and MXM, respectively. No statistically significant difference was observed between the DC and MXM pigment inhibition potential (*p*-value 0.522 and 0.502, respectively).

**Figure 5:**
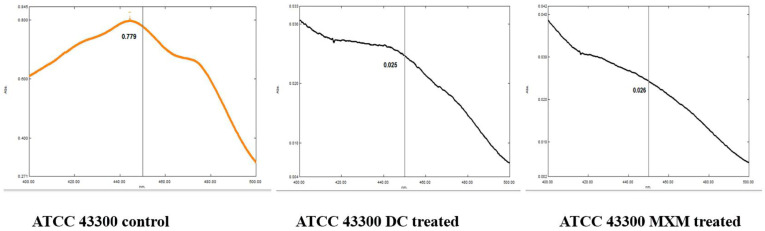
Absorbance spectra of STX extracted from standard strain MRSA before and after DC/MXM treatment.

By observing the spectral profile of the STX extract of standard strain ATCC 43300, the control isolate revealed an OD_450_ of 0.779 before treatment, followed by a remarkable decrease in the absorbance reading after DC/MXM treatment, reaching 0.025 and 0.026 for DC and MXM, respectively (Figure 5).

### 2.7. Effect of Staphyloxanthin Inhibition on Protease Production 

The pigmented isolates treated with DC/MXM showed larger inhibition zone diameters than untreated isolates. In contrast, the inhibition zone diameters of non-pigmented isolates were mostly unaffected by the treatment. 

**Figure 6:**
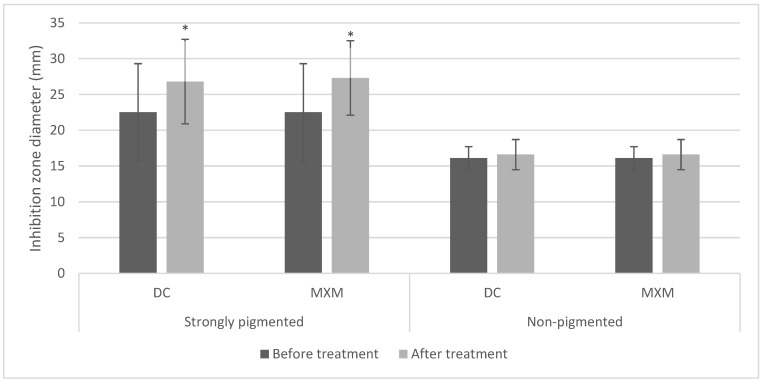
Bar chart representing mean and standard deviation values for inhibition zones of protease activity. Asterisks indicate statistical significance at *p* ≤ 0.05.

These findings were further confirmed by the repeated measures ANOVA test, which revealed a statistically significant increase in inhibition zones after implementing DC/MXM treatment on the STX producers, with *p*-values < 0.001. However, non-pigmented isolates showed no statistically significant change in inhibition zones after using both drugs, with *p*-values of 0.655 (Figure 6). Whether with strongly or non-pigmented isolates, there was no statistically significant difference between the inhibition zones of the two drugs (*p*-value 0.834 and 1 for DC and MXM respectively).

### 2.8. Effect of Staphyloxanthin Inhibition on DNase Activity

**Figure 7:**
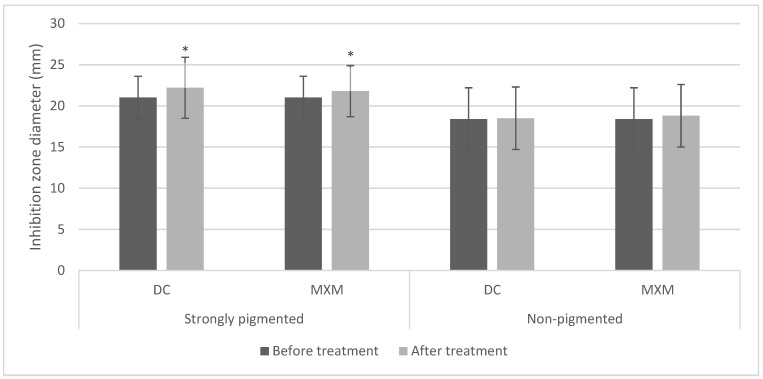
Bar chart representing mean and standard deviation values for inhibition zones of nuclease activity. Asterisks indicate statistical significance at *p* ≤ 0.05.

For the STX producers, most of the treated isolates had shown an enhancement in DNase production. Conversely, most of the non-pigmented isolates showed no change in the measurement of the inhibition zone diameter in comparison to their controls. Moreover, the standard strain responded to the treatment with an enhanced potential for nuclease production. The statistical analysis using the repeated measures ANOVA test revealed a statistically significant increase in inhibition zones after treating STX producers with DC/MXM (*p*-values < 0.001 and 0.014 for DC and MXM, respectively). On the other hand, there was no statistically significant change in inhibition zones after using both drugs on STX non-producers (*p*-values 0.749 and 0.205 for DC and MXM, respectively) (Figure 7). No statistically significant difference was observed between DC and MXM nuclease activity-enhancing potential in the strongly pigmented as well as non-pigmented isolates (*p*-value 0.806 and 0.854 for DC and MXM respectively).

### 2.9. ERIC-PCR Analysis

**Figure 8:**
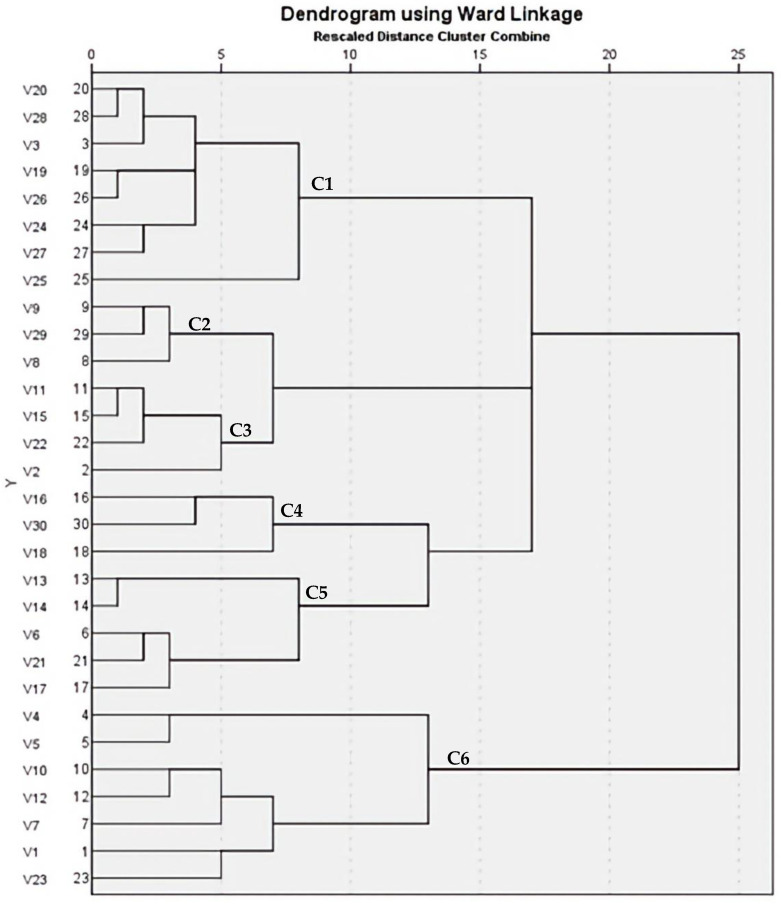
Dendrogram of 30 MRSA isolates showing genetic heterogeneities.

The variable banding pattern of ERIC-PCR gel electrophoresis revealed diversity among the 30 MRSA isolates (15 strongly pigmented and 15 non-pigmented isolates). The DNA bands yielded from REP-PCR type amplification were thoroughly analyzed, and a phylogenetic tree for the isolated strains was designed by the GelClust to reveal a wide range of genetic heterogeneities among the selected isolates. The cluster analysis and related dendrogram is shown in Figure 8. Based on the results shown in Figure 8, the MRSA isolates were categorized into two groups that were furtherly subdivided to six clusters (C1–C6) with a discriminatory power of 0.8368, which is closer to 1.0 than 0, revealing wide heterogeneities among the sources of the tested isolates. Dendrogram analysis revealed an overall similarity of 65.75% among the six clusters. Furthermore, the similarity between each C1–C6 cluster member was 61.5, 80, 83.5, 41.5, 62.3, 65.75%, respectively.

### 2.10. Detection of mecA Gene among MRSA Isolates

Among the 30 tested isolates, 100% were found to be *mec*A positive confirming methicillin resistance by disc diffusion method.

### 2.11. DC/MXM Treatment Effect on Virulent Genes Expression 

**Figure 9:**
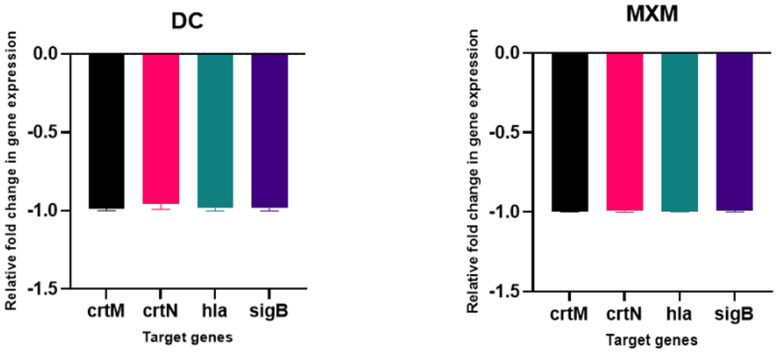
The influence of DC/MXM treatment (at sub-MICs of 47 µg/mL and 59 µg/mL for DC and MXM, respectively) on the expression of candidate genes involved in STX synthesis and virulence factors production when compared with the expression of the housekeeping gene 16S ribosomal RNA. Assays were performed in triplicates, and error bars indicate SDs from the mean. A *p-*value of <0.05 is considered statistically significant.

**Figure 10:**
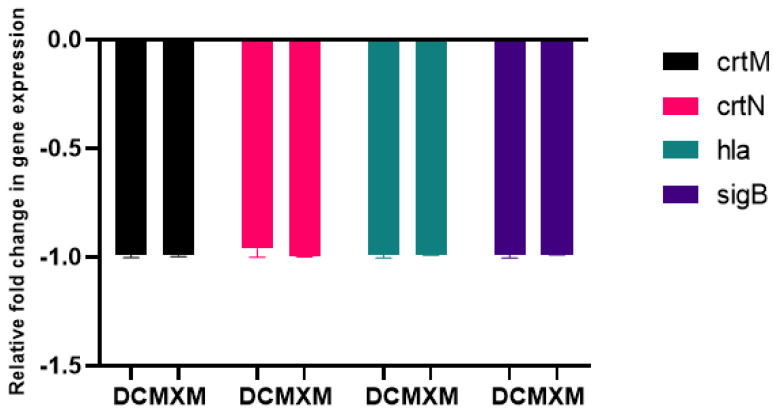
Comparing the effects of DC treatment vs. MXM treatment (at their sub-MICs of 47 µg/mL and 59 µg/mL for DC and MXM respectively) on the level of virulent gene expression. Error bars indicate SDs from the mean.

The results of the qPCR analysis validated the downregulation of target virulence genes expression, such as *crt*M, *crt*N, *hla* and *sig*B, upon DC/MXM treatment in comparison to untreated cells (Figure 9). DC caused an inhibition of target-gene expression of 99.4, 96, 98.25 and 98.6% in the treated cells in comparison with untreated cells for the previously mentioned genes, respectively. As for MXM, gene expression reduction reached 99% for all target genes in comparison to untreated cells. When comparing the effect of DC and MXM at their sub-MICs on gene expression, as shown in Figure 10, no significant difference was found between the different treatments, with *p-*values of 0.965, >0.999, 0.233 and >0.999 and for *crt*M, *crt*N, *hla* and *sig*B genes, respectively.

### 2.12. Molecular Docking 

**Figure 11:**
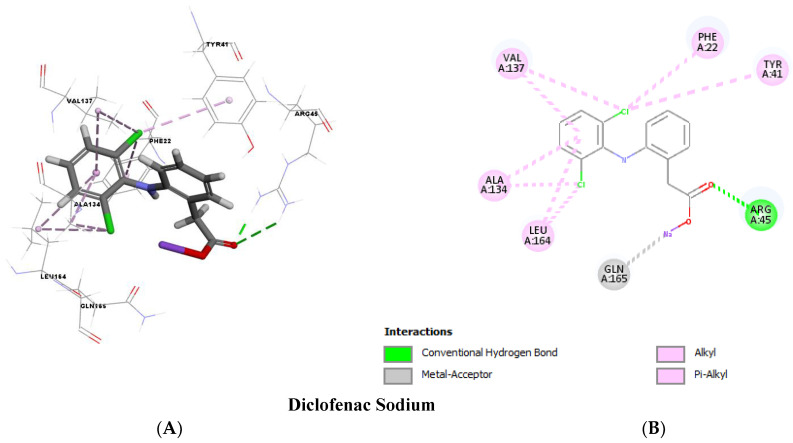
Molecular docking analysis showing (Three-dimensional) 3D (**A**) and (two-dimensional) 2D (**B**) representation of interaction patterns of DC with CrtM receptor.

**Figure 12:**
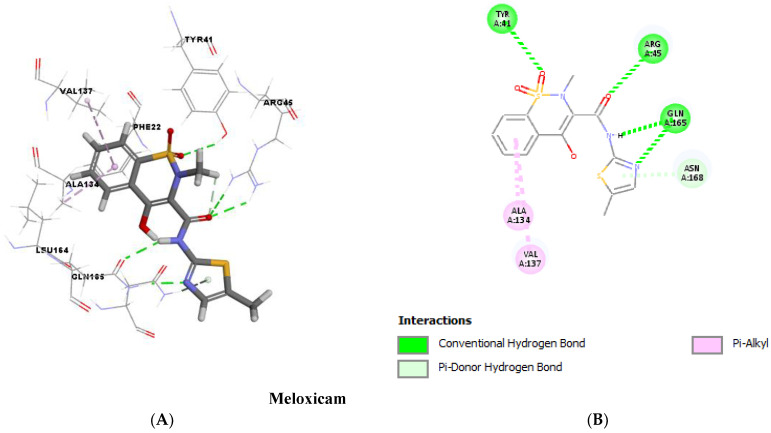
Molecular docking analysis showing (Three-dimensional) 3D (**A**) and (two-dimensional) 2D (**B**) representation of interaction patterns of MXM with CrtM receptor.

Molecular docking analysis, as shown in Figure 11 and Figure 12, validated the ability of both DC and MXM to bind to the CrtM protein of MRSA. Both drugs were found to efficiently bind to the enzyme with several hydrogen bonds and hydrophobic bonds. Molecular modeling results are presented in Table 2. The results demonstrate the ability of both drugs to interact using strong hydrogen bonds with CrtM binding sites and their potential activity as inhibitors to this target.

## 3. Discussion

For many years, antibiotic resistance has been a global concern among the nations. Egypt has an alarmingly high prevalence of MRSA in the healthcare setting, threatening the well-being of its population [20]. This notorious pathogen has been imposing a heavy financial burden on the society due to ineffective treatment regimens by conventional antimicrobials [21]. Introducing anti-pathogenic agents that target virulence factors without affecting bacterial growth could be a potential approach to control MRSA infectivity. The present study highlights the role of MXM as compared to DC as anti-virulence agents targeting STX inhibition. The phenotypic detection of *S. aureus* isolates revealed that all isolates were cefoxitin resistant, confirming methicillin resistance. Similar findings were reported by Abbas et al. [15]. However, Ibrahim et al. reported 81% methicillin resistance among their isolates [22]. Detection of the *mec*A gene by PCR is considered the gold standard method for confirming methicillin resistance among *S. aureus* strains. Several studies, including the current work, revealed the concordance of cefoxitin disc diffusion results with the presence of the *mec*A gene; this can be attributed to cefoxitin inducing the potential for *mec*A expression [23].

All the studied isolates were susceptible to vancomycin and linezolid. The results were consistent with Shady et al. (91% vancomycin susceptibility) and Alfeky et al. (92% linezolid susceptibility) [19,24]. Despite the emergence of vancomycin intermediate and resistant strains, vancomycin remains the mainstay for treating serious MRSA infections. Linezolid however could be a suitable alternative in the case of vancomycin resistance [25]. The current study revealed 36.25% clindamycin resistance among the tested isolates. Similarly, Kishk et al. reported a 38.6% resistance rate to clindamycin among their isolates [26]. This work supports the fact that fluoroquinolones resistance is common among MRSA isolates, where 41.25% of the isolates showed resistance to ciprofloxacin. The results are in line with Hashem et al.’s findings in 2013 which reported a rate of 57.7% resistance and Alseqely et al. who reported the highest resistance rate in 2021, reaching 96%, which can be attributed to the prevalence of *spa* type t037 among MRSA isolates (which is moxifloxacin resistant) [27,28]. However, in agreement with Abbas et al. [21] where 30% of the isolates were MDR, the findings were lower than that reported by Rasmi et al. (54.2%) [29]. The increasing rate of resistance could be due to the high consumption of ciprofloxacin and clindamycin in treating MRSA infections. As compared to Abbas et al. [21], in our study, the DC was selected as a reference drug owing to its potent previously proven anti-STX effect based on Abass et al. [21], and we used it in order to compare its results to another promising anti-inflammatory drug (MXM) whose anti-STX potential had not yet been investigated. On the other hand, this study aimed to confirm the DC inhibitory activity on STX production and link this inhibitory effect to the production of other important virulence determinants of MRSA clinical isolates. We tested the influence of both drugs both phenotypically (protease and nuclease as well as hemolysin toxin activities) and genotypically (analyzing the level of gene expression using PT-PCR, *crt*M, *crt*N and global transcriptional regulator *sig*B along with the *hla* gene coded for hemolysin) on the biosynthesis of STX, which was reflected in the decrease of the sum of the major virulence characteristics of the MRSA clinical isolates. Both DC and MXM effects were also investigated through molecular docking analysis for further confirmation. DC and MXM potently inhibited the synthesis of STX at 47 and 59 µg/mL to reach 79.3–98% and 80.6–96.7% inhibition, respectively. 

Roughly 90% of *S. aureus* have been reported to be STX producers in previous studies, giving the human pathogen an enhanced bacterial resistance against innate immunity and a persistence in target organs [14]. Our findings are in agreement with the previous studies, since the majority of the isolates (62.5%) were STX producers, conforming to Zhang et al.’s results that revealed 59% STX producers among their isolates [30]. Recent studies have highlighted the inhibitory ability of some FDA-approved drugs against *S. aureus* virulence [10,14,31,32]. The present study clearly demonstrates the ability of DC and MXM to hinder STX production in MRSA isolates. The inhibition of this hallmark virulence factor lends support to for the potential role of anti-inflammatories as adjuvants to conventional antimicrobial therapy. The investigated MIC of DC and MXM was found to be 375 and 973.5 µg/mL respectively. The anti-virulence activity of the tested drugs was studied at their sub-MICs (47 and 59 µg/mL for DC and MXM respectively) to avoid possible bactericidal effects. This approach was adopted by many recent studies on anti-virulence therapy [33,34,35,36]. The carotenoid pigment inhibition by DC and MXM was evaluated phenotypically to reveal 79.3–98% and 80.6–96.7% inhibition potential in treated cells for DC and MXM, respectively. In line with our findings, the Selvaraj et al. study on myrtenol showed an inhibition rate of 20–65%, and Abbas et al. reported the glyceryl trinitrate inhibitory potential to be 63.37–70.98% on the STX production of treated isolates [33,37]. 

The current study revealed an enhanced activity of matrix-degrading enzymes such as protease and nuclease upon STX inhibition by DC/MXM treatment, agreeing with a recent study conducted by Valliammai et al. [35]. The results may be attributed to the up regulation of the *agr* system by DC/MXM that in turn impedes biofilm formation and promotes the expression of degrading enzymes [38]. To confirm the diversity of the studied clinical isolates, ERIC PCR fingerprint analysis was performed and yielded six clusters of thirty tested MRSA isolates. Previous studies suggest the importance of discriminatory power calculation when analyzing *S. aureus,* due to the abundance of repetitive DNA elements among their strains [39]. Hence, our study proposes a discriminatory power (D value) of 0.8368, which is closer to 1.0 than it is to 0.0, suggesting the ability of the typing method to distinguish each member of the strain population from all other members of that population [40]. 

Both qPCR analysis and molecular docking were employed to demonstrate the molecular mechanism responsible for STX inhibition. Treated cells with both tested drugs revealed a significant repression of *crt*M and *crt*N genes encoding for the primary enzymes in STX biosynthetic pathway as well as the *crt*OPQMN operon regulator *sigB,* confirming the phenotypic results of the spectrophotometric analysis. Molecular docking was performed to test the validity of CrtM as a potential target for anti-virulence therapy, since it has been reported by many studies as an efficient drug target to inhibit STX synthesis [33,35,41]. Both drugs revealed a high binding energy to CrtM through strong hydrogen bonds. The binding energies of previously reported CrtM inhibitors such as celastrol, hesperidin and carvacrol were found to be less than that reported by DC or MXM [36,41,42], which further validates the STX inhibitory potential of DC/MXM treatment. Regarding the other virulent genes’ expression, DC/MXM treatment had interestingly downregulated the expression of the major virulence gene *hla* encoding for α-hemolysin production. In a similar manner, myrtenol and diflunisal repressed the expression of *hla* gene in treated cells [33,43]. 

## 4. Materials and Methods

### 4.1. Bacterial Strains

A total of eighty clinical isolates of *S. aureus* were obtained from the Clinical Microbiology laboratory of two different hospitals in Egypt. The isolates were recovered from various clinical specimens of unidentified patients, including blood, pus, nasal swabs, wound swabs, and groin swabs from hospital routine checkup. The *crt*M mutant *S. aureus* strain and Wild type (W.T) *S. aureus* strain were kindly provided by Dr. George Y. Liu (Division of Pediatric Infectious Diseases and the Immunology Research Institute, California, USA). Standard MRSA strain ATCC 43300 (*mec*A positive *S. aureus*) was also included in this study. The isolates were identified by conventional and genetic identification methods, as previously reported [44]. The study was conducted in accordance with the Declaration of Helsinki and approved by the Ethics Committee of Faculty of Pharmacy, Ain Shams University (ENREC-ASU-2021-) (Ethics approval committee No: RHDIRB2020110301 REC # 116).

### 4.2. Stock Solutions

To prepare the 2000 µg/mL diclofenac stock solution, 0.2 g of diclofenac sodium (DC) (EIPICO, Cairo, Egypt) was dissolved in 100 mL of distilled water along with 1.5 mL of dimethyl sulfoxide (DMSO) (Sigma-Aldrich, St. Louis, MO, USA). Meloxicam (MXM) ampoules (10,000 µg/mL) were purchased from Medical Union Pharmaceuticals Co. (MUP, Cairo, Egypt).

### 4.3. Antibiotic Susceptibility of Clinical Isolates 

The Kirby–Bauer disc diffusion method was used to determine the susceptibility of the clinical isolates to the antimicrobial agents Cefoxitin (30 µg), Linezolid (30 µg), Ciprofloxacin (5 µg) and Clindamycin (2 µg). The antibiotic discs were purchased from Hi-media (Mumbai, India). It was carried out according to the Clinical and Laboratory Standards Institute guidelines 2020 [45] and as previously described in [34]. The Minimum inhibitory concentration (MIC) of Vancomycin against *S. aureus* isolates was determined by the standard micro-dilution method according to the procedures outlined by the guidelines of the CLSI 2020 [45] and as previously described in [46].

### 4.4. Staphyloxanthin Production Assay

The production of STX by MRSA isolates was evaluated according to Valliammai et al., with some modifications. The isolates were cultured overnight in tryptic soy broth (TSB; Thermo Fisher Scientific, Waltham, MA, USA), and the suspensions were adjusted to 1 McFarland (approximately 3 × 10^8^ CFU/mL) then swabbed on the surface of tryptic soy agar (TSA; Thermo Fisher Scientific, Waltham, MA, USA) plates supplemented with 1.5% glycerol-monoacetate to enhance the production of the carotenoid pigment [47]. The plates were incubated at 37 °C for 48 h and were observed for STX production and photographed [34].

### 4.5. Determination of MICs of Tested Drugs

The MICs of DC and MXM were determined by broth micro-dilution methods according to the procedures outlined by the guidelines of the CLSI 2020 [45]. The pigmented bacterial isolates were selected, and the procedure was conducted as described by [46]. The results were recorded, and the MIC was determined. In order to rule out the possibility of the inhibitory effects on bacterial growth, trials using sub-MICs of the tested drugs (1/4, 1/8 and 1/16) were implemented, and bacterial growth was compared with untreated bacterial isolates [21].

### 4.6. Staphyloxanthin Inhibition Assay 

The STX extraction and quantification was performed according to Al-kazaz et al. [48]. The most appropriate sub-MICs that inhibited STX without affecting bacterial growth were selected. TSA plates (supplemented with 1.5% Glycerol-monoacetate) with and without the sub-MICs of DC or MXM were inoculated with bacterial suspensions of 1 McFarland. After 48 h of incubation, TSA plates were washed thrice with distilled water. The resulting suspensions were centrifuged at 8000 rpm for 15 min. The pellets were mixed with 3 mL of 99% methanol and heated in a water bath at 55 °C for 30 min with gentle stirring, then cooled for 10 min and centrifuged again at 8000 rpm for 15 min. The absorbance of the supernatant was measured spectrophotometrically at 450 nm [48]. The control isolates (without treatment) were divided according to their optical density into three groups (strongly, weakly and non-pigmented) [46]. The reduction in STX production for treated isolates was expressed in percentages in relation to untreated isolates according to the following formula: percentage of inhibition = [(Control OD_450_ nm − Treated OD_450_ nm)/Control OD_450_ nm] × 100 [21].

### 4.7. Extracellular Protease Assay 

The change in proteolytic activity of MRSA isolates was evaluated using skimmed milk agar. The skimmed milk agar was prepared by adding fresh skimmed milk to sterile molten nutrient agar (BD Difco, Newark，NJ, USA) to a final concentration of 5% *v*/*v* [31]. Overnight cultures of MRSA isolates in TSB with and without sub-MICs of tested drugs were centrifuged at 6000 rpm for 10 min. The wells were made into the skimmed milk media and filled with 100 μL of the supernatants and incubated at 37 °C for 24 h. The clear zones around the wells were measured [32]. This assay was carried out in duplicate, and average reading of inhibition zones was recorded. 

### 4.8. Extracellular Nuclease Assay 

The nuclease activity alterations were detected using DNase agar (Oxoid, Hampshire, UK) according to Lagacé-Wiens et al. with some modifications. Overnight cultures of treated (with sub-MICs of the tested drugs) and untreated MRSA isolates in TSB were centrifuged at 6000 rpm for 10 min, then the supernatants (100 μL aliquots) were injected into cups made in DNase agar plates. The DNase agar plates were then incubated for 24 h at 37 °C [49]. The clear zones surrounding the cups indicating DNase activity were measured and recorded [35]. This assay was carried out in duplicate, and average reading was recorded.

### 4.9. Determination of Genetic Diversity Using ERIC-PCR

Genotyping of the MRSA isolates was performed by Enterobacterial Repetitive Intergenic Consensus Polymerase Chain Reaction (ERIC-PCR) using a pair of published primers (F: 5′-ATG TAA GCT CCT GGG GAT TCA C-3′) (R: 5′-AAG TAA GTG ACT GGG GTG AGC-3′) [50]. The PCR master mix was prepared according to the instructions of Emerald Amp GT PCR mastermix (Takara Bio INC, Japan) Code No. RR310A kit. The thermal cycling protocol was performed as follows: initial denaturation of the target DNA sequence at 94 °C for 5 min, followed by 35 cycles of secondary denaturation at 94 °C for 30 s, annealing at 52 °C for 1 min, extension of the primers by thermostable polymerase at 72 °C for 2 min and a final extension step for 12 min at 72 °C, followed by cooling to 4 °C. Electrophoresis of PCR products was done using 1.5% agarose gel. The ERIC fingerprinting data were transformed into a binary code depending on the presence or absence of each band. Dendrograms were generated by the unweighted pair group method with arithmetic average (UPGMA) and Ward’s hierarchical clustering routine. Cluster analysis and dendrogram construction were performed with SPSS version 22 (IBM 2013) [40]. The discriminatory index (D-value) was calculated using an online discriminatory power calculator (http://insilico.ehu.es/mini_tools/discriminatory_power/) (accessed on 10 December 2022). The similarity index (Jaccard/Tanimoto Coefficient and number of intersecting elements) between all samples was calculated using an online tool (https://planetcalc.com/1664/) (accessed on 10 December 2022) [51].

### 4.10. Detection of mecA Gene by cPCR

The genotypic identification of the MRSA isolates was conducted by conventional PCR for detection of *mec*A gene using a pair of primers (F: 5′-GTA GAA ATG ACT GAA CGT CCG ATA-3′) (R: 5′-CCA ATT CCA CAT TGT TTC GGT CTA A-3′) [52]. The PCR master mix was prepared as mentioned under Section 4.9. The PCR cycling conditions were as follows: initial denaturation of the target DNA sequence at 94 °C for 5 min, followed by 30 cycles of secondary denaturation at 94 °C for 30 s, annealing of both forward and reverse primers was at 50 °C for 30 s, extension by thermostable polymerase was at 72 °C for 30 s and a final extension step for 2 min at 72 °C was done [23].

### 4.11. Real-Time PCR (qPCR) Analysis

The qPCR analysis was performed to assess the effect of DC/MXM treatment on selected genes encoding for STX biosyntheis in MRSA, such as *crt*M and *crt*N, and those involved in virulence, such as *hla* (alpha-hemolysin) and *sig*B (global transcriptional regulator). Total RNA was extracted from DC/MXM treated and untreated isolates after 24 h of incubation by the Trizol method. Conversion into cDNA and qPCR analysis was performed in a 1-step reaction using GoTaq^®^ 1-Step RT-qPCR System A6020 (Promega, Madison, WI, USA) as per the manufacturer’s protocol. The qPCR data were analyzed using QuantStudio 5 (Fisher scientific, Madison, WI, USA). Expression of the selected genes was quantified by 2^(–ΔΔCt)^ method after the normalization of cycle threshold (Ct) values of all tested genes to that of the housekeeping gene *16srRNA* [36]. The primer sequences of the tested genes are listed in Table 3.

### 4.12. Molecular Docking Analysis

Molecular docking was performed to assess the binding energy and interactions of DC and MXM with CrtM of MRSA. The 3D crystal structure of CrtM (ID: 2ZCQ) was downloaded from the Protein Data Bank. The 3D structures of the target compounds used in the study were sketched using the BIOVIA Discovery Studio v 4.5 program (PubChem ID: 5018304 and 54677470 for DC and MXM, respectively). The docking step was performed using the cDOCKER protocol, in which the scoring system was based on the cDOCKER energy. The 3D and 2D structures were visualized through BIOVIA Discovery studio visualizer 2016 v16.1.0.15350 [53]. 

### 4.13. Statistical Analysis 

Numerical data were explored for normality by checking the distribution of data and by using tests of normality (Kolmogorov–Smirnov and Shapiro–Wilk tests). All the experiments were carried out in triplicates. Data were presented as median, range, mean and standard deviation (SD) values. The repeated measures ANOVA test and Mann–Whitney U test were implemented. The significance level was set at *p* ≤ 0.05. Statistical analysis was performed with IBM SPSS Statistics for Windows, Version 23.0. Armonk, NY, USA: IBM Corp.

## 5. Conclusions

The current study unveiled the anti-virulence potential of DC/MXM treatment against MRSA. Both drugs revealed significant STX inhibition that was further confirmed by molecular docking analysis, emphasizing the strong interaction between the CrtM binding site and DC/MXM. Additionally, real-time PCR analysis highlighted the efficiency of the proposed treatment in downregulating the expression of genes responsible for STX synthesis, along with the transcriptional regulator gene *sig*B and other virulence genes such as the *hla* gene. Genotypic analysis was in accordance with phenotypic analysis, with no significant difference between the anti-virulence potential of both drugs. Thus, the present study proposes the repurposing of DC/MXM as adjuvants to conventional antimicrobial treatments against serious MRSA infections. 

## Figures and Tables

**Table 1:** Antimicrobial resistance pattern among clinical isolates.

Antimicrobial Agents	Susceptibility Pattern of the Clinical Isolates, (N = 80)					
	Sensitive (S)		Intermediate (I)		Resistant (R)	
	N	%	N	%	N	%
Cefoxitin	0	0	0	0	80	100
Clindamycin	50	62.5	1	1.25	29	36.25
Ciprofloxacin	44	55	3	3.75	33	41.25
Linezolid	80	100	0	0	0	0
Vancomycin	80	100	0	0	0	0

N: number of the isolates.

**Table 2:** Molecular modeling results.

STX Inhibitors	cDOCKER Energy (Kcal/mol)	cDOCKER Interaction Energy (Kcal/mol)	Interactions with Amino Acid Residues of CrtM	
Diclofenac	−28.5335	−36.6979	Hydrogen bonds	Hydrophobic interactions
			2 H-bonds with ARG45	8 hydrophobic bonds with PHE22, TYR41, ALA134, VAL137 and LEU164
Meloxicam	−23.4162	−40.4063	6 H-bonds with TYR41, ARG45, GLN165 and ASN168	2 hydrophobic bonds with ALA134 and VAL137

**Table 3:** List of primers used for qPCR analysis.

Target Gene	Primer Sequence (5′–3′)
*crt*M	F-CTGCTAATTCTATGATTGGTTGTGC
	R-TGGGAATATTATGCAGCTATMGCAG
*crt*N	F-GATGAAGCTTTGACGCAACA
	R-TTCGCATGATACGTTTGCTC
*hla*	F-GAA AGG TAC CAT TGC TGG TCA
	R-AAG GCC AGG CTA AAC CAC TT
*sig*B	F-AAG TGA TTC GTA AGG ACG TCT
	R-TCG ATA ACT ATA ACC AAA GCC T

*crt*M coded for dehydrosqualene synthase, *crt*N coded for 4,4-diapophytoene desaturase, *hla* coded for alpha-hemolysin, *sig*B coded for a global transcriptional regulator.

## Data Availability

All the data supporting the findings are included in the manuscript.
